# Effects of Uric Acid-Lowering Treatment on Glycemia: A Systematic Review and Meta-Analysis

**DOI:** 10.3389/fendo.2020.00577

**Published:** 2020-09-02

**Authors:** Juan Chen, Jing Ge, Min Zha, Jun-Jun Miao, Zi-Lin Sun, Jiang-Yi Yu

**Affiliations:** ^1^Department of Endocrinology, Jiangsu Province Hospital of Chinese Medicine, Affiliated Hospital of Nanjing University of Chinese Medicine, Nanjing, China; ^2^Department of Endocrinology, Zhongda Hospital, Institute of Diabetes, Medical School, Southeast University, Nanjing, China

**Keywords:** uric acid, blood glucose, glycated hemoglobin A1c, systematic review, meta-analysis

## Abstract

**Background:** Serum uric acid levels have been shown to be associated with increased risk of diabetes. However, it remains unclear whether uric acid-lowering therapy (ULT) is associated with improved glycemic status. This study aimed to summarize evidence from randomized controlled trials (RCTs) to investigate whether ULT reduces fasting blood glucose (FBG) and glycated hemoglobin A1c (HbA1c) levels.

**Methods:** PubMed, Embase, and the Cochrane Library were searched from inception until April 10, 2019. Moreover, in order to maximize the search for articles on the same topic, the reference lists of included studies, relevant review articles and systematic reviews were reviewed. Parallel RCTs investigating the effect of ULT on FBG or HbA1c levels were considered for inclusion. An English language restriction was applied. Data were screened and extracted independently by two researchers. Meta-analyses were performed using random-effects models to calculate the weighted mean differences (WMDs) and 95% confidence intervals (CIs).

**Results:** Four trials with 314 patients reported the effect of ULT with allopurinol on FBG and 2 trials with 141 patients reported the effect of ULT with allopurinol on HbA1c. Treatment with allopurinol resulted in a significant decrease in FBG (WMD: −0.61 mmol/L, 95% CI: −0.93 to −0.28), but only a trend of reduction in HbA1c (WMD: −0.47%, 95% CI: −1.16 to 0.22). Notably, the subgroup analyses showed that treatment with allopurinol was associated with reduced FBG levels in patients without diabetes (WMD: −0.60 mmol/L, 95% CI: −0.99 to −0.20), but not in patients with diabetes. In addition, the dose of allopurinol treatment ≥200 mg daily resulted in a reduction of FBG levels (WMD: −0.59 mmol/L, 95% CI: −0.95 to −0.23), whereas low-dose allopurinol (<200 mg daily) had no effect on FBG levels.

**Conclusions:** The findings suggest that ULT with allopurinol may be effective at reducing glycemia, but such an improvement does not appear to be observed in patients with diabetes. The findings require confirmation in additional trials with larger sample sizes.

## Introduction

As early as 1981, uric acid has been recognized as a powerful chemical antioxidant, which may play a major role in lengthening life-span and decreasing age-specific cancer rates due to its proposed antioxidant properties (1, 2). However, high serum uric acid above the normal range is deleterious. Since the prevalence of hyperuricemia is increasing (3, 4), the role of it in health and disease has gained much more attention. Accumulating evidences have demonstrated that uric acid induces oxidative damage in a variety of tissues (5, 6). Furthermore, hyperuricemia has been identified as an independent risk factor for various chronic conditions such as hypertension, diabetes, cardiovascular disease, metabolic syndrome, and chronic kidney disease (7–11). It has been recently reported an association between elevated uric acid and a hepatokine that acts as a molecular link between non-alcoholic fatty liver disease and metabolic disorders (12). Moreover, a strong association of elevated uric acid levels with increased risk for cardiovascular mortality has been demonstrated (13). For each 59.48 μmol/L increase in serum uric acid, the cardiovascular mortality increases by a 28% (14). Accordingly, uric acid is considered as a potential therapeutic target for improving health outcomes. Subsequently, there is a growing interest in exploring the influence of uric acid-lowering therapy (ULT) on the above-mentioned chronic conditions. Currently, studies have indicated that ULT can prevent the increase in blood pressure (15, 16), delay the progression of kidney disease (17, 18), and reduce the risk of cardiovascular disease and mortality (19). However, it remains unclear whether ULT reduces the risk of diabetes or glycemic parameters.

Studies have reported that serum uric acid is significantly and positively correlated with fasting and 2-h plasma glucose in subjects with impaired glucose tolerance, as well as in subjects with normal glucose tolerance (20, 21). In addition, there are evidences that every 1 mg/dL increase in uric acid is significantly associated with a 0.082 mg/dL increase in fasting plasma glucose in individuals with prediabetes (22), and a 20% increase in the risk of type 2 diabetes (23). Therefore, the use of serum uric acid concentration has been suggested as a marker for assessing the risk of future incident type 2 diabetes (24). Moreover, it has also been reported that uric acid is positively associated with glycated hemoglobin A1c (HbA1c) in individuals with normal glucose tolerance (21). However, the positive associations between uric acid and glycemic parameters no longer exist once the disease progresses to the stage of diabetes, instead, turn into negative correlations (21, 25, 26). As early as three decades ago, study has showed that uric acid is obviously higher in individuals with prediabetes, whereas lower in individuals with diabetes compared with those with normal glucose tolerance (27). It seems that there are some differences in the interaction of uric acid and blood glucose in different glucose tolerance conditions. Nevertheless, it cannot be denied that uric acid plays an important role in the onset of diabetes (8), especially recent studies have indicated a significant role of uric acid on the development of insulin resistance, a major characteristic in the pathogenesis of type 2 diabetes (24, 28). Uric acid may serve as a potential biomarker of deterioration in glucose metabolism.

Therefore, ULT may be beneficial for the improvement of blood glucose. Yet limited studies, until now, have reported the influence of ULT on glycemic parameters. In addition, evidences from human intervention studies are controversial (29, 30). Thus, in this study, we aimed to assess the effect of ULT on glycemia control, which may be instructive for clinical practice.

## Methods

This study was conducted in accordance with the Preferred Reporting Items for Systematic Reviews and Meta-Analyses (31).

### Data Sources

PubMed, Embase and the Cochrane Library were searched for eligible trials from the earliest publication date available through April 10, 2019. Moreover, in order to maximize the search for articles on the same topic, the reference lists of included studies, relevant review articles and meta-analyses were screened. Only parallel randomized controlled trials (RCTs) in English were considered for inclusion. The terms allopurinol, febuxostat, probenecid, sulfinpyrazone, benzbromarone, rasburicase, pegloticase, uricosuric agent, antigout agent, xanthine oxidase inhibitor, uricase, urate oxidase, urate lowering therapy, gout suppressant, uric acid lowering therapy, blood glucose, plasma glucose, serum glucose, HbA1c, glycated hemoglobin A1c, glycosylated hemoglobin A1c (HbA1c) were searched alone or in combination. The details of the search strategy are available in the Supplementary Material.

### Study Selection

The titles and abstracts were screened for relevance by two independent investigators (JC and JG). After being identified as relevant articles, the full texts were individually assessed by both investigators, independently, to determine whether the article was qualified for eligibility criteria. Disagreements occurred between the investigators were resolved through consultation with a third investigator (J-YY). If suitable data were not available in the published papers, the corresponding authors were contacted to obtain this information. Studies were considered eligible if they (1) were parallel RCTs; (2) compared any uric acid-lowering agent at any dose with no uric acid-lowering agent (defined as placebo or no treatment); (3) provided information on any of the prespecified primary outcomes that had to be reported pre- and post-intervention; (4) were published in English.

### Data Extraction

The major demographic and clinical data from the included RCTs were extracted by two independent investigators (JC and JG). The data extracted from each study included author name, publication year, baseline characteristics of participants, sample size, medication used, follow-up duration, and study outcomes (including blood glucose or HbA1c at baseline and post-intervention). Disagreements were resolved by discussion between two investigators or consulting a third investigator (J-YY) if necessary. Risk of bias assessment was performed by two independent investigators (JC and JG) using the Cochrane Collaboration risk of bias tool (32). The included studies were assessed for sequence generation, allocation concealment, blinding, detection bias, attrition bias, and reporting bias. Each item was assessed as low, unclear or high risk of bias with supportive data from the study. Discrepancies were resolved by discussion and consensus, with a third investigator if necessary.

### Data Synthesis and Statistical Analysis

The statistical analyses were undertaken using STATA (version 15.0, College Station, Texas). The changes from baseline to endpoint were calculated for assessing the impact of ULT on blood glucose and HbA1c (32). Heterogeneity was evaluated using the *Q* test, with the value <0.1 indicative of the existence of heterogeneity and the *I*^2^ statistic, with the value >50% indicative of significant heterogeneity. The weighted mean differences (WMDs) with 95% confidence intervals (CIs) were calculated using random-effects models for all analyses, which could provide more conservative results than fixed-effects models (32). Subgroup analyses were conducted to investigate the impact of potential moderating factors on glycemic parameters as follows: (1) the presence or absence of diabetes; (2) dose of allopurinol (>200 mg daily or <200 mg daily); (3) treatment duration (>3 months or <3 months); (4) age of participants (>55 years old or <55 years old). Sensitivity analyses were performed to evaluate the robustness of our findings. A 2-sided *P* < 0.05 was set for statistical significance.

## Results

### Study Selection and Study Characteristics

**Figure 1:**
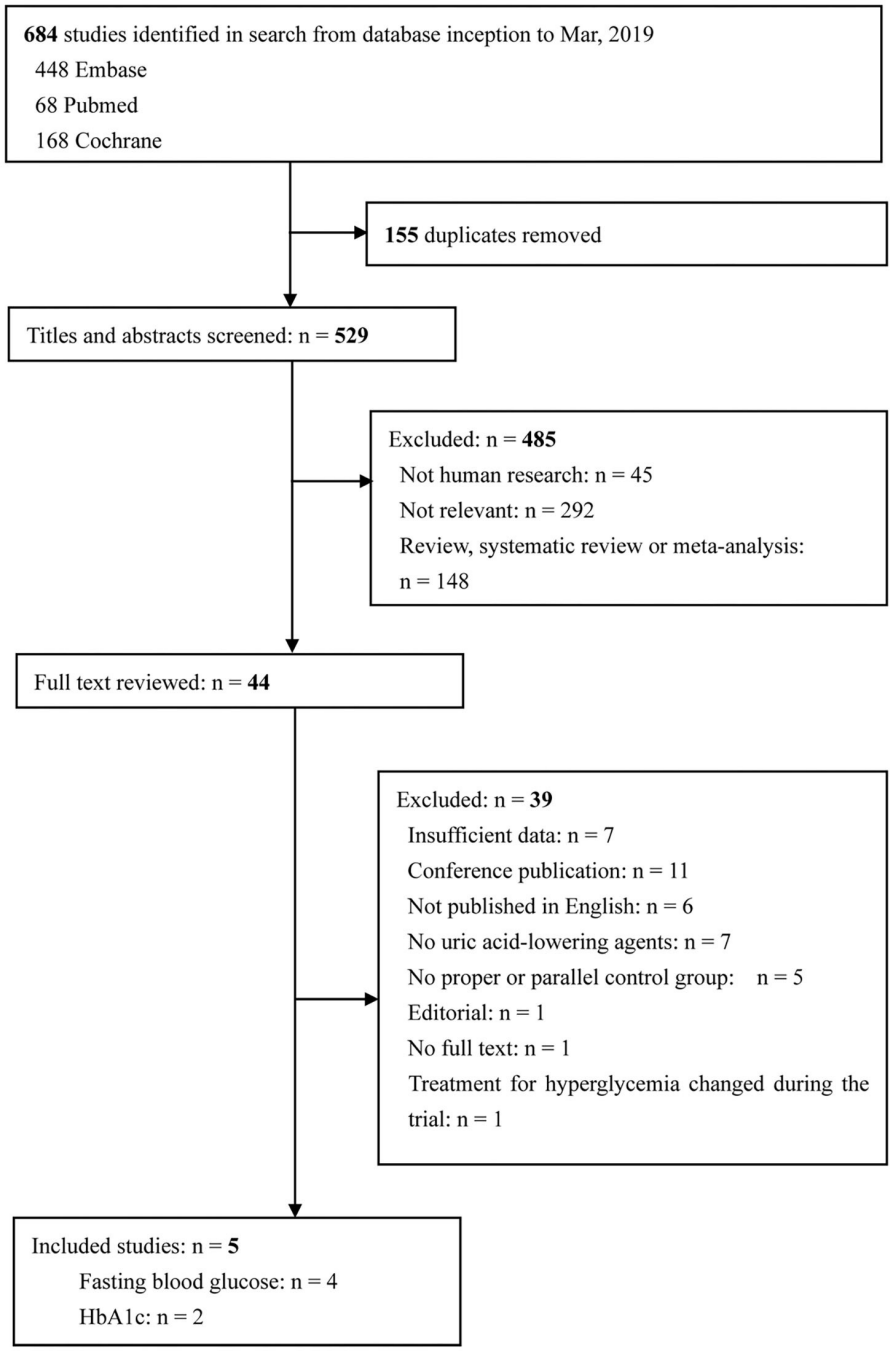
Flow diagram of study selection. Finally, five articles were included in the present study. Among these articles, three researches provided data on FBG, one research provided data on HbA1c, and another research provided both FBG and HbA1c data. FBG, fasting blood glucose; HbA1c, glycated hemoglobin A1c.

The study selection process and search result are shown in Figure 1. In total, 684 articles were identified, with 448 articles identified in Embase, 68 articles identified in Pubmed, 168 articles identified in the Cochrane Central Register of Controlled Trials. After excluding 155 duplicates, 529 potentially eligible articles were selected. Of these articles, 485 were excluded because they were not human research (*n* = 45), were irrelevant to our present meta-analysis (*n* = 292), or were review, systematic review or meta-analysis (*n* = 148). The remaining 44 articles were searched for further evaluation. Finally, four and two papers were included in the meta-analyses that evaluated FBG and HbA1c levels, respectively. There were no changes in medication use throughout the intervention periods in all included studies.

The selected studies were published from 2004 to 2017 (29, 30, 33–35). Study sample sizes ranged from 40 to 100, with a total of 354 randomized patients, including 180 patients in the intervention group and 174 in the control group. Allopurinol was used as ULT in all included studies. The daily dose of allopurinol varied between 100 mg and 900 mg. The durations of allopurinol treatment ranged from 2 weeks to 4 months. Three trials investigated the use of allopurinol in participants with diabetes, 1 trial in acute coronary syndrome, and 1 trial in asymptomatic hyperuricemia. The detailed characteristics of the studies included in the meta-analysis are presented in Table 1.

**Table 1:** Baseline characteristics of included studies.

**Study**	**Patients,** **No**.	**Mean age, year**	**Male, %**	**Type of patient population**	**DM duration, years**	**Experimental intervention**	**Baseline of UA (μmol/L)**	**Baseline** **of HbA1c (%)**	**Baseline** **of BG (mmol/L)**
Afshari et al. (29)	ULT: 20 PC: 21	ULT: 49.05 PC: 50.63 Total: 49.9	31	Diabetic patients	ULT: 7.76 PC: 6.35	Allopurinol 300 mg daily for 2 weeks	N/D	ULT: 9.10 ± 0.79 PC: 7.01 ± 0.50	ULT: 8.61 ± 1.69 PC: 8.31 ± 0.97
Huang et al. (35)	ULT: 50 PC: 50	56.3	60	Acute coronary syndrome, non-diabetes subjects	N/A	Allopurinol 600 mg daily during ACS acute phase and followed by 200 mg daily for 1 month	ULT: 584.2 ± 35.6 PC: 583.6 ± 34.2 Total:583.9	N/D	ULT: 5.4 ± 0.7 PC: 5.3 ± 0.6
Momeni et al. (33)	ULT: 20 PC: 20	ULT: 56.3 PC: 59.1 Total: 57.8	45	Diabetic patients	ULT: 11.8 PC: 13.3	Allopurinol 100 mg daily for 4 months	ULT: 354.6 ± 72.0 PC: 386.8 ± 130.9 Total: 370.7	N/D	ULT: 9.91 ± 4.44 PC: 8.41 ± 3.43
Dogan et al. (30)	ULT: 50 PC: 50	ULT: 50.5 PC: 50.0 Total: 50.3	51	Diabetic patients	ULT: 4.4 PC: 4.1	Allopurinol 900 mg daily for 4 months	ULT: 297.5 ± 47.6 PC: 285.6 ± 65.45 Total: 291.6	ULT: 6.1 ± 2.1 PC: 6.2 ± 1.5	N/D
Takir et al. (34)	ULT: 40 PC: 33	ULT: 52.15 PC: 49.88 Total: 51.1	49	Asymptomatic hyperuricemia, non-diabetes subjects	N/A	Allopurinol 300 mg daily for 3 months	ULT: 467.7 ± 36.9 PC: 443.3 ± 53.6 Total: 456.7	N/D	ULT: 5.7 ± 0.50 PC: 5.17 ± 0.46

*ULT, uric acid-lowering therapy; PC, placebo or control; N/A, not applicable; N/D, not determined; DM, diabetes mellitus; UA, uric acid*.

### Risk of Bias and Publication Bias

Results of the quality assessments performed by assessing the risk for bias across a range of domains are shown in the Supplementary Table 1. Of the five included studies, 3 were at low risk of bias and 2 were at high risk. Because only five studies were included in our meta-analysis, a linear regression test of funnel plot asymmetry (Egger's test) could not be performed.

### Primary Outcome: FBG and HbA1c

**Figure 2:**
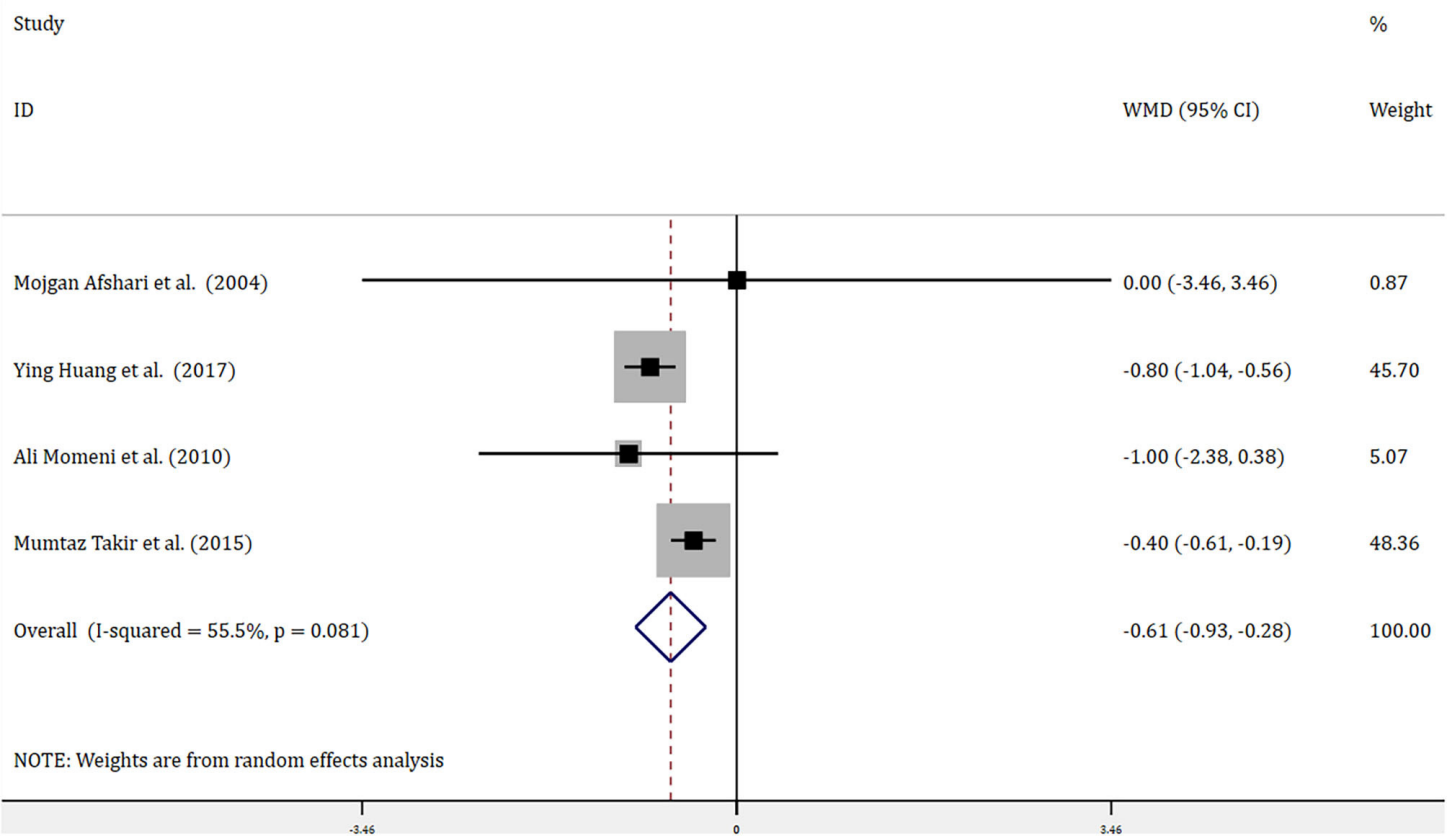
Forest plot of meta-analysis of the effect of ULT with allopurinol on FBG. Data are pooled WMDs with 95% CIs. ULT, uric acid-lowering therapy; FBG, fasting blood glucose; WMD, weighted mean difference; CIs, confidence intervals.

**Figure 3:**
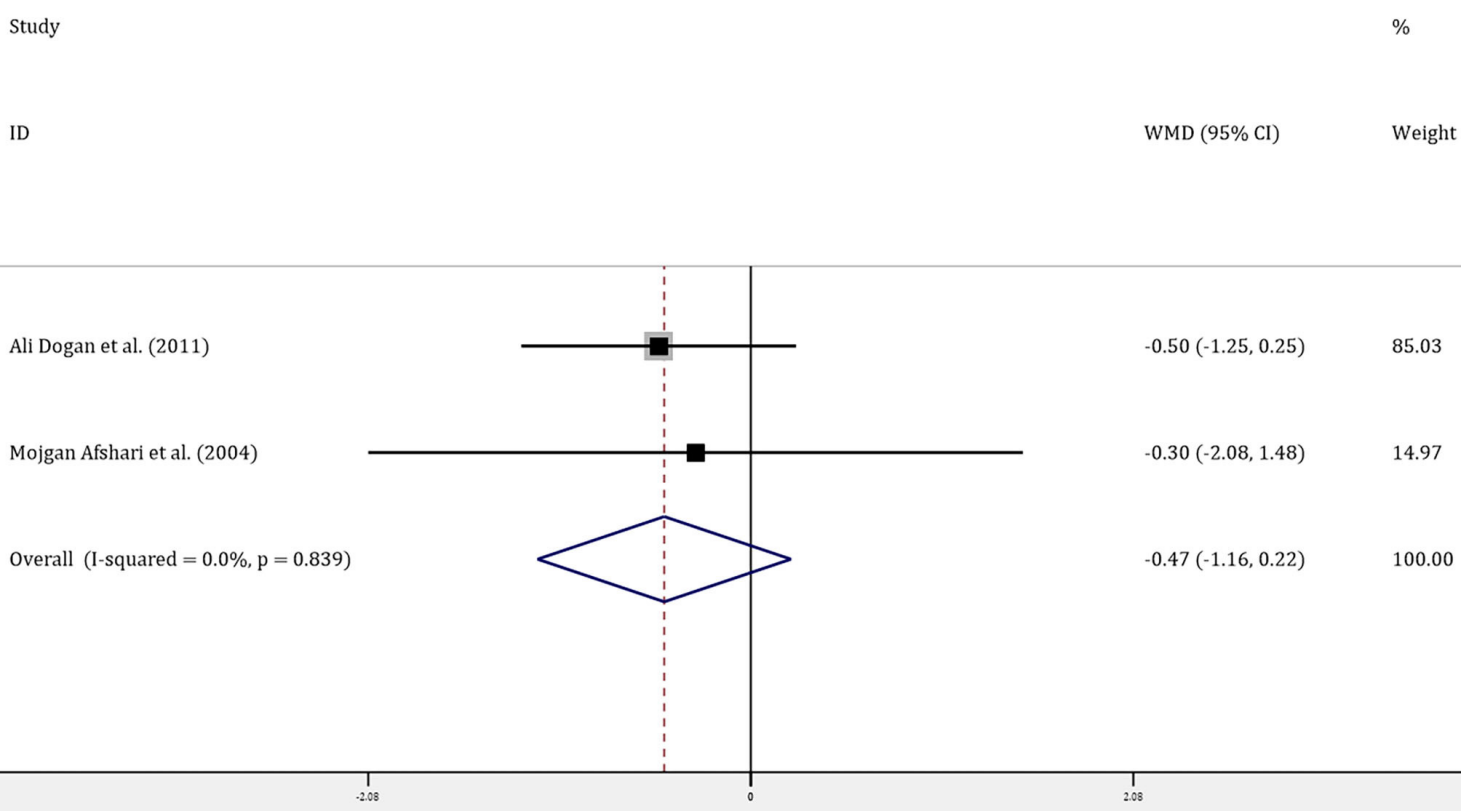
Forest plot of meta-analysis of the effect of ULT with allopurinol on HbA1c. Data are pooled WMDs with 95% CIs. ULT, uric acid-lowering therapy; HbA1c, glycated hemoglobin A1c; WMD, weighted mean difference; CIs, confidence intervals.

Four trials with 314 patients reported the effects of allopurinol on FBG and 2 trials with 141 patients reported the effects of allopurinol on HbA1c. Forest plots for the overall effect of treatment on FBG and HbA1c are presented in Figures 2, 3, respectively. For FBG, the pooled WMD was −0.61 (95% CI: −0.93 to −0.28). Since the evidence collected in our meta-analysis showed heterogeneity (*I*^2^ = 55.5%), a random-effects model was performed. The *z*-test result for overall effects was statistically significant (*P* < 0.001), indicating a significantly greater reduction in FBG in patients treated with allopurinol than those in the control group. However, for HbA1c, the pooled WMD was −0.47 (95% CI: −1.16 to 0.22), with low heterogeneity (*I*^2^ = 0). The z-test result for overall effects showed no statistical significance (*P* = 0.18), which suggested that treatment with allopurinol did not significantly impact HbA1c.

### Sensitivity Analysis

To evaluate the robustness of our conclusions, sensitivity analysis was performed. As shown in the Supplementary Figure 1, the association between treatment with allopurinol and FBG did not change considerably after exclusion of any one study from the analysis, indicating that the pooled results were reliable.

### Subgroup Analysis

Significant reduction in FBG levels was observed in association with treatment with allopurinol in the subgroup of patients without diabetes (WMD −0.60, 95% CI: −0.99 to −0.20, *P* = 0.003, *I*^2^ = 84.1%). However, analysis of patients with diabetes (*n* = 141) showed no evidence of benefit for FBG (WMD −0.86, 95% CI: −2.15 to 0.42, *P* = 0.187, *I*^2^ = 0). A further subgroup analysis was undertaken according to the dose of allopurinol treatment. The data showed that the dose of intervention >200 mg daily resulted in an improvement of FBG levels (WMD −0.59, 95% CI: −0.95 to −0.23, *P* = 0.001, *I*^2^ = 68.7%), whereas the dose <200 mg daily had no effect on FBG levels. In addition, when taking the length of allopurinol intervention into consideration, significant reduction of FBG was observed in patients receiving allopurinol regardless of whether the treatment duration was >3 months or <3 months (WMD −0.80, 95% CI: −1.03 to −0.56, *P* < 0.001, *I*^2^ = 0; WMD −0.41, 95% CI: −0.62 to −0.21, *P* < 0.001, *I*^2^ = 0, respectively). In addition, obviously reduced FBG levels were found in the subgroup of patients with age <55 years old (WMD −0.40, 95% CI: −0.61 to −0.19, *P* < 0.001, *I*^2^ = 0), as well as in patients with age >55 years old (WMD −0.81, 95% CI: −1.04 to −0.57, *P* < 0.001, *I*^2^ = 0). Detailed results of the subgroup analysis are shown in Table 2.

**Table 2:** Subgroup analysis of the association of ULT with allopurinol with FBG.

**Meta-analysis and subgroup analysis of FBG**	**No. of study**	**No. of subject**		**FBG (95% CIs)**	***P*-value**
		**Allopurinol**	**Control**		
**Diabetes**					
Yes	2	70	71	−0.86 (−2.15 to 0.42)	0.187
No	2	90	83	−0.60 (−0.99 to −0.20)	0.003
**Dose of intervention**					
<200 mg	1	50	50	−1.00 (−2.38 to 0.38)	0.156
≥200 mg	3	110	104	−0.59 (−0.95 to −0.23)	0.001
**Length of treatment**					
<3 months	2	70	71	−0.80 (−1.03 to −0.56)	<0.001
≥3 months	2	90	83	−0.41 (−0.62 to −0.21)	<0.001
**Age**					
<55 years	2	60	54	−0.40 (−0.61 to −0.19)	<0.001
≥55 years	2	100	100	−0.81 (−1.04 to −0.57)	<0.001

*ULT, uric acid-lowering therapy; FBG, fasting blood glucose; CIs, confidence intervals*.

## Discussion

### Summary

Our analyses found that ULT with allopurinol was significantly associated with reduced FBG, but no evidence of HbA1c reduction by treatment with allopurinol. In addition, subgroup analyses revealed that treatment with allopurinol reduced FBG levels in participants without diabetes. However, no significant effect on FBG was observed in those with diabetes. Moreover, the results also suggested that the high dose of allopurinol treatment, >200 mg daily, might be more effective than low dose of allopurinol treatment in reducing FBG since no significant decrease in FBG levels was observed at dose <200 mg daily. Nevertheless, the result regarding dose should be interpreted with caution as only 1 study was available for inclusion in the low-dose group. Furthermore, ULT with allopurinol led to a significant improvement in FBG regardless of whether the treatment duration was >3 months or <3 months. Moreover, the beneficial effects of ULT on FBG were observed in patient receiving allopurinol whether the age was <55 years old or >55 years old. It seems likely that age and length of allopurinol intervention might have no impact on this effect, which, albeit, still requires further investigations.

### Comparison With Existing Literature

To date, a number of epidemiologic studies have reported that prediabetics have significantly higher serum uric acid levels than non-diabetics (27, 36). In addition to people with pre-diabetes, a close relationship between uric acid levels and FBG was also observed in non-diabetic individuals (25). Furtherly, studies suggested that uric acid plays an important role in the onset of diabetes (25, 36). Therefore, people may benefit from ULT, especially those with hyperuricemia. Although a lot of researches focus on the correlation between uric acid and diabetes, there are few studies concerned about the efficacy of ULT on blood glucose. In addition, studies conducted on this topic have resulted in inconsistent findings. Takir et al. found ULT with allopurinol was associated with a significant improvement in FBG (34). However, another study found that there was no significant differences observed in changes in FBG (37). In the present study, we found that lowering uric acid led to an obvious improvement in FBG. Additionally, our study found a non-significant decrease in HbA1c after lowering uric acid. However, it is noteworthy that there were only two studies with small sample size that explored the influence of lowering uric acid on HbA1c, which might affect the outcomes of interest.

Furthermore, based on our subgroup analyses, the improvement in FBG in response to lowing uric acid was observed in patients without diabetes, but not in patients with diabetes. Noteworthy, previous studies have found that compared with non-diabetics, diabetics have obviously lower serum uric acid levels (27, 36). Different from non-diabetics, the correlation between uric acid levels and FBG changed from positive correlation to negative correlation in diabetic individuals (25). Moreover, serum uric acid has been reported to fall with increasing duration of the diabetes (36). Our previous study also suggested that with increasing blood glucose, enhanced concentration of glucose in the lumen of the proximal convoluted tubule inhibited uric acid reabsorption, leading to decreased uric acid (26). Therefore, ULT may not cause a significant change in FBG in patients with diabetes since they have obviously lower serum uric acid levels. The effect of ULT on blood glucose seems to be weakened when disease progresses to the stage of diabetes. Further studies are necessary to clarify how do uric acid interact with blood glucose in different glucose tolerance conditions including impaired fasting glucose and impaired glucose tolerance. Since hyperuricemia has been identified as an independent risk factor for diabetes, the effect of ULT on the incidence of diabetes is also worth being explored in future studies.

In addition, the significant decrease in FBG levels observed at the dose of allopurinol treatment ≥200 mg daily suggested that high dose of allopurinol might be superior to low dose in improving FBG. A recent study by Mizukoshi et al. also found significant decline in HbA1c levels in patients treated with high dose topiroxostat, while the levels of HbA1c did not change in patients with low dose topiroxostat (38). The effect on improving glycemia may be owing to the ability of high doses of uric acid-lowering medication in reducing oxidative stress (39). Nevertheless, in the present study, the result regarding dose should be interpreted with caution as only one study was available for inclusion in the low-dose group. It will be important that future research assess the association of intervention dose with glycemic parameters. Moreover, our study showed that age and length of allopurinol intervention did not affect the ability of ULT in improving FBG. Our meta-analysis only included a very limited number of researches, thereby interpreting the results should be more cautious. More researches are in need to determine whether age and treatment duration are potential moderating factors.

### Implications for Research and Practice

One of the most revealing findings of this meta-analysis was that ULT with allopurinol was significantly associated with reduced FBG. This is of clinical importance for patients with hyperuricemia, since every 1 mg/dl increase in uric acid was correlated with an estimated 20% risk of type 2 diabetes according to previous report (23). Although such an improvement in FBG in response to lowing uric acid was observed in patients without diabetes, but not in patients with diabetes, it cannot be ignored that uric acid has a significant impact on the development of hyperglycemia.

Although not fully understood, it is speculated that the observed beneficial effects of ULT on FBG might be underlined by the following mechanisms. Study has demonstrated that the addition of uric acid activates hepatic gluconeogenesis by reducing adenosine monophosphate-activated protein kinase activity (40), suggesting that ULT may contribute to the improvement of hyperglycemia. In addition to the liver, adipose tissue has been confirmed to have abundant xanthine oxidoreductase activity (41). Previous study noted that uric acid induces oxidative stress via activating nicotinamide adenine dinucleotide phosphate oxidase and increasing reactive oxygen species production in adipocytes (5). It is worth noting that oxidative stress in the adipose tissue has been recognized as a major cause of insulin resistance (41). Moreover, it has also been confirmed that lowering uric acid can improve insulin resistance in obese mice (42). In addition, there is evidence that high uric acid inhibits pancreatic β cells proliferation and glucose-induced insulin secretion, while increases the production of reactive oxygen species (6). The study pointed out that uric acid may contribute to abnormal glucose metabolism by inducing oxidative stress. Taken together, individuals treated with ULT more prone to have deceased FBG may explained by reduced hepatic gluconeogenesis and improved insulin resistance. Future studies are needed to validate the result of our study and clarify whether ULT reduces FBG by above-mentioned mechanisms.

### Strengths and Limitations

This meta-analysis has some limitations. First, until now, few studies have been designed to on the association between ULT and glycemic parameters. In our meta-analysis, all included studies used allopurinol as uric acid-lowering medication. However, it is unknown that whether patients can still benefit from other uric acid-lowering medication such as febuxostat, probenecid, and benzbromarone. It is necessary for us to do further research on this topic. Second, our study had a language restriction, which may cause some selection bias. Third, since our meta-analysis pooled only small RCTs, the results should be interpreted with caution. Future RCTs with larger sample size and higher quality are necessary to investigate the effect of ULT on glycemic parameters. Despite the aforementioned limitations, the present study could still provide valuable information to help us understand the effect of ULT on blood glucose.

## Conclusions

In conclusion, this meta-analysis suggests that ULT with allopurinol may be effective at reducing glycemia. However, such an improvement does not appear to be observed in patients with diabetes. The findings require confirmation in additional trials with larger sample sizes.

## Data Availability Statement

All datasets generated for this study are included in the article/Supplementary Material.

## Author Contributions

J-YY had full access to all of the data in the study, take responsibility for the integrity of the data, and the accuracy of the data analysis. JC, JG, Z-LS, and J-YY contributed to the study concept and design. JC, JG, J-JM, and MZ contributed to the acquisition, analysis, and interpretation of the data. JC and JG drafted the manuscript and performed the statistical analyses. JC, JG, J-JM, MZ, Z-LS, and J-YY critically revised the manuscript. All authors reviewed the manuscript and approved the final manuscript.

## Conflict of Interest

The authors declare that the research was conducted in the absence of any commercial or financial relationships that could be construed as a potential conflict of interest.
