# Supplementary material for: Effects of Uric Acid-Lowering Treatment on Glycemia: A Systematic Review and Meta-Analysis
Source: Front Endocrinol (Lausanne). 2020 Sep 2;11:577. doi: 10.3389/fendo.2020.00577 (PMC7493655; doi:10.3389/fendo.2020.00577)

Search Strategy

**PubMed**

1. allopurinol [Text Word]
2. febuxostat [Text Word]
3. probenecid [Text Word]
4. sulfinpyrazone [Text Word]
5. benzbromarone [Text Word]
6. rasburicase [Text Word]
7. pegloticase [Text Word]
8. 1 or 2 or 3 or 4 or 5 or 6 or 7
9. uricosuric agent [Text Word]
10. antigout agent [Text Word]
11. xanthine oxidase inhibitor [Text Word]
12. uricase [Text Word]
13. urate oxidase [Text Word]
14. urate lowering therapy [Text Word]
15. gout suppressant [Text Word]
16. uric acid lowering therapy [Text Word]
17. 9 or 10 or 11 or 12 or 13 or 14 or 15 or 16
18. 8 or 17
19. glucose [Text Word]
20. Glycated Hemoglobin A1c [Text Word]
21. Glycosylated Hemoglobin A1c [Text Word]
22. HbA1c [Text Word]
23. 19 or 20 or 21 or 22
24. 18 and 23
25. random*[Text Word]
26. blind*[Text Word]
27. 25 or 26
28. 24 and 27

**Embase**

1. 'allopurinol'/exp OR allopurinol OR 'febuxostat'/exp OR febuxostat OR 'probenecid'/exp OR probenecid OR 'sulfinpyrazone'/exp OR sulfinpyrazone OR 'benzbromarone'/exp OR benzbromarone OR 'rasburicase'/exp OR rasburicase OR 'pegloticase'/exp OR pegloticase

2. 'uricosuric agent'/exp OR 'uricosuric agent' OR (('uricosuric'/exp OR uricosuric) AND ('agent'/exp OR agent)) OR 'antigout agent'/exp OR 'antigout agent' OR (antigout AND ('agent'/exp OR agent)) OR 'xanthine oxidase inhibitor'/exp OR 'xanthine oxidase inhibitor' OR (('xanthine'/exp OR xanthine) AND ('oxidase'/exp OR oxidase) AND ('inhibitor'/exp OR inhibitor)) OR 'uricase'/exp OR uricase OR 'urate oxidase'/exp OR 'urate oxidase' OR (('urate'/exp OR urate) AND ('oxidase'/exp OR oxidase)) OR 'urate lowering therapy'/exp OR 'urate lowering therapy' OR (('urate'/exp OR urate) AND lowering AND ('therapy'/exp OR therapy)) OR 'gout suppressant' OR (('gout'/exp OR gout) AND suppressant) OR 'uric acid lowering therapy' OR (uric AND ('acid'/exp OR acid) AND lowering AND ('therapy'/exp OR therapy))

3. 1 OR 2

4. 'glucose'/exp OR glucose OR 'glycated hemoglobin a1c'/exp OR 'glycated hemoglobin a1c' OR (glycated AND ('hemoglobin'/exp OR hemoglobin) AND a1c) OR 'glycosylated hemoglobin a1c'/exp OR 'glycosylated hemoglobin a1c' OR (glycosylated AND ('hemoglobin'/exp OR hemoglobin) AND a1c) OR 'hba1c'/exp OR hba1c

5. 3 AND 4

6. random* OR blind*

7. 5 AND 6

**Cochrane Library**

1. allopurinol [All Text]
2. febuxostat [All Text]
3. probenecid [All Text]
4. sulfinpyrazone [All Text]
5. benzbromarone [All Text]
6. rasburicase [All Text]
7. pegloticase [All Text]
8. 1 or 2 or 3 or 4 or 5 or 6 or 7
9. uricosuric agent [All Text]
10. antigout agent [All Text]
11. xanthine oxidase inhibitor [All Text]
12. uricase [All Text]
13. urate oxidase [All Text]
14. urate lowering therapy [All Text]
15. gout suppressant [All Text]
16. uric acid lowering therapy [All Text]
17. 9 or 10 or 11 or 12 or 13 or 14 or 15 or 16
18. 8 or 17
19. glucose [All Text]
20. Glycated Hemoglobin A1c [All Text]
21. Glycosylated Hemoglobin A1c [All Text]
22. HbA1c [All Text]
23. 19 or 20 or 21 or 22
24. 18 and 23
25. random*[All Text]
26. blind*[ All Text]
27. 25 or 26
28. 24 and 27

| Author, year | Random sequence generation | Allocation concealment | Blinding of participants and personnel | Blinding of outcome assessment ^a^ | Incomplete outcome data addressed | Selective reporting |
| --- | --- | --- | --- | --- | --- | --- |
| Ying Huang et al. 2017 | Low | Unclear | High | Low | Low | Low |
| Ali Momeni et al. 2010 | Low | Unclear | Low | Low | Low | Low |
| Ali Dogan et al.  2011 | Low | Unclear | Low | Low | Low | Low |
| Mojgan Afshari et al. 2004 | Low | Unclear | Low | Low | Low | Low |
| Mumtaz Takir et al. 2015 | Low | Unclear | High | Low | Low | Low |
| ^a^ The outcome assessment for blood glucose or HbA1c is objective, which is not likely to be affected by lacking of blinding. | | | | | | |

**TABLE1** | Risk of Bias Assessment.

**FIGURE 1**| Sensitivity analysis.


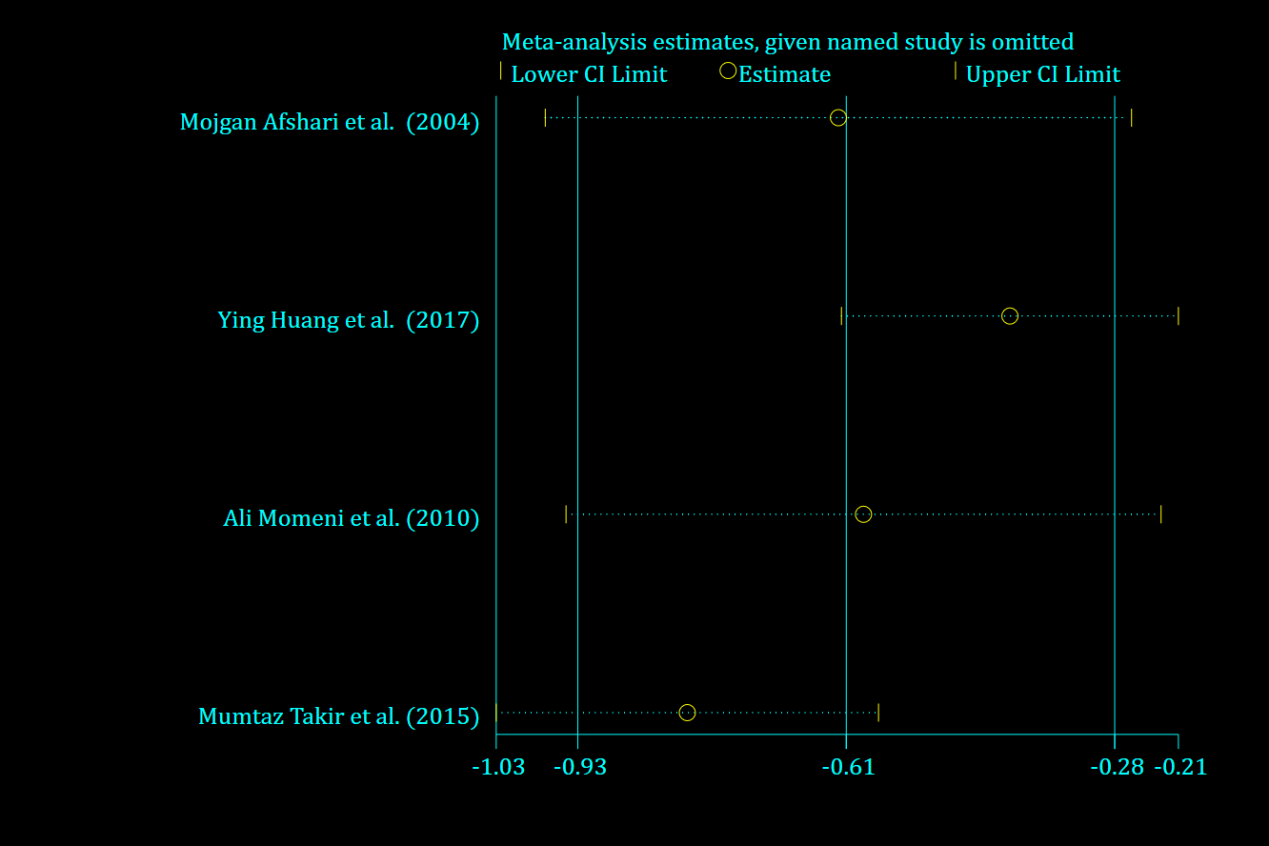

Supplement: Supplementary file 1 [file Table_1.DOCX]
